# Troubleshooting Gait Disturbances in Parkinson’s Disease With Deep Brain Stimulation

**DOI:** 10.3389/fnhum.2022.806513

**Published:** 2022-05-16

**Authors:** Nicoló G. Pozzi, Chiara Palmisano, Martin M. Reich, Philip Capetian, Claudio Pacchetti, Jens Volkmann, Ioannis U. Isaias

**Affiliations:** ^1^Department of Neurology, University Hospital of Würzburg and Julius Maximilian University of Würzburg, Würzburg, Germany; ^2^Parkinson’s Disease and Movement Disorders Unit, IRCCS Mondino Foundation, Pavia, Italy; ^3^Parkinson Institute Milan, ASST Gaetano Pini-CTO, Milan, Italy

**Keywords:** Parkinson’s disease, freezing of gait (FOG), deep brain stimulation (DBS), subthalamic nucleus (STN), globus pallidus pars interna (GPi), pedunculopontine nucleus (PPN)

## Abstract

Deep brain stimulation (DBS) of the subthalamic nucleus or the globus pallidus is an established treatment for Parkinson’s disease (PD) that yields a marked and lasting improvement of motor symptoms. Yet, DBS benefit on gait disturbances in PD is still debated and can be a source of dissatisfaction and poor quality of life. Gait disturbances in PD encompass a variety of clinical manifestations and rely on different pathophysiological bases. While gait disturbances arising years after DBS surgery can be related to disease progression, early impairment of gait may be secondary to treatable causes and benefits from DBS reprogramming. In this review, we tackle the issue of gait disturbances in PD patients with DBS by discussing their neurophysiological basis, providing a detailed clinical characterization, and proposing a pragmatic programming approach to support their management.

## Introduction

In Parkinson’s disease (PD) a progressive dopaminergic neuronal loss alters the functioning of the cortico-striatal-thalamic network and determines an increasing motor impairment (Albin et al., 1989; Isaias et al., 2006, 2011, 2012; Litvak et al., 2011; de Hemptinne et al., 2013; Cagnan et al., 2015). Along with disease progression, PD leads to increasing disability with worsening of quality of life (Rascol et al., 2011). One of the main determinants of poor quality of life in PD is gait impairment, mainly because it correlates with mobility reduction, falls and hospitalization (Muslimović et al., 2008).

The term gait impairment is unspecific and encompasses a variety of gait disturbances that range from shuffling gait to walking difficulties due to dyskinesias. PD can also present peculiar gait disturbances, such as freezing of gait (FOG) (Nutt et al., 2011a). This clinical variability reflects a complex and diverse pathophysiology that challenges an appropriate treatment, which remains limited at best (Muslimović et al., 2008). Dopaminergic replacement therapy may indeed yield only a partial benefit and eventually deteriorate some aspects of gait and balance in PD (Peterson and Horak, 2016), possibly because of the unselective impact of levodopa on the locomotor network (Curtze et al., 2015; Palmisano et al., 2020a).

Deep brain stimulation (DBS) of the subthalamic nucleus (STN-DBS) or the globus pallidus pars interna (GPi-DBS) is an established treatment for PD that can provide a marked improvement of quality of life in PD patients with motor fluctuations (The Deep-Brain Stimulation for Parkinson’s Disease Study Group, 2001; Deuschl et al., 2006; Follett et al., 2010; Schuepbach et al., 2013). Comparative studies showed a similar benefit for the two targets (Follett et al., 2010; Tagliati, 2012; Weaver et al., 2012; Williams et al., 2014; Ramirez-Zamora and Ostrem, 2018) with motor improvement lasting for more than 30 years for STN-DBS (Merola et al., 2011) and over 10 years for GPi-DBS (Mansouri et al., 2018). Despite this sustained improvement of motor symptoms, the effect of DBS on gait impairment remains debated.

Converging evidence showed a positive effect for STN-DBS and GPi-DBS on gait in the first year after surgery (Bakker et al., 2004), while long-term follow up studies reported a progressive worsening of gait for both targets (Pötter-Nerger and Volkmann, 2013). A meta-regression analysis of 12 studies (nine with STN-DBS and three with GPi-DBS) on postural instability and gait disorder (PIGD) in PD showed that PIGD worsens to the preoperative state already 2 years after STN-DBS in meds-on condition (i.e., with medication) (St George et al., 2010). In line with these data, up to 42% of PD patients with STN-DBS report a subjective worsening of gait performance 6 months after surgery, despite general motor improvement (van Nuenen et al., 2008).

While chronic progressive loss of efficacy might be due to disease progression and to concomitant worsening of postural control (Limousin and Foltynie, 2019), an early gait deterioration after DBS is likely related to suboptimal stimulation (Farris and Giroux, 2013; Pötter-Nerger and Volkmann, 2013; Limousin and Foltynie, 2019). In line with this hypothesis, a case series review of 50 PD patients with PIGD and unsatisfactory STN-DBS outcomes showed that suboptimal stimulation was responsible for up to 52% of cases and reprogramming of DBS parameters improved the clinical outcome in 75% of cases (Farris and Giroux, 2013). Still, DBS programming is an iterative and poorly standardized process that requires expertise and careful trial-and-error adjustments (Kühn and Volkmann, 2017).

In this review, we will tackle this issue and provide a pragmatic troubleshooting programming approach to manage early gait disturbances in PD patients with DBS. We will first describe the pathophysiological mechanism of gait impairment in PD, then provide a clinical characterization of gait disturbances and finally discuss the possible stimulation alternatives.

A comprehensive discussion of the long-term effects of DBS on PIGD is beyond the scope of this review and can be found elsewhere (Fasano et al., 2015; Limousin and Foltynie, 2019). Likewise, gait disturbances arising directly after DBS surgery are usually related to surgical causes and have already been reviewed elsewhere (Adams et al., 2011; Fleury et al., 2016; Sketchler and Shahed, 2019).

## The Human Supraspinal Locomotor Network

In recent years, technological advances allowed to obtain important information about the physiology and pathophysiology of human gait, revealing the complex neural architecture of the locomotor network (Takakusaki et al., 2004; Takakusaki, 2017; Pozzi et al., 2019). This network comprises the primary motor cortex, the supplementary motor area (SMA), the basal ganglia, the thalamus, the mesencephalic locomotor region (MLR) with the pedunculopontine nucleus (PPN) and the cuneiform nucleus (CN), the cerebellum and the spinal network of central pattern generator (CPGs) (la Fougère et al., 2010; Tard et al., 2015; Snijders et al., 2016; Takakusaki, 2017).

The rhythmic activity of CPGs generates stepping movements, which are initiated and modulated by the supraspinal locomotor network (for review Nutt et al., 2011b).

The MLR is the core of locomotor adaptation as it is essential for the integration of sensorimotor and emotional stimuli that modifies the patterned activity of CPGs (Collomb-Clerc and Welter, 2015; Takakusaki, 2017). The main anatomical structures of the MLR are the CN and the PPN, which together regulate posture, muscular tone, and locomotion initiation (Takakusaki, 2008, 2017). A detailed discussion of the brainstem control of posture and gait is reported in Takakusaki (2008, 2017). In brief, the glutamatergic CN neurons exert a prokinetic effect possibly starting locomotion by releasing the CPGs, while the GABAergic PPN neurons inhibit the activity of the SNr that suppresses locomotor activities (Takakusaki, 2008, 2017). The PPN is innervated from the basal ganglia (in particular, the STN and the GPi), the thalamus (parafascicular and center-median nucleus) and the motor and premotor cortices (e.g., supplementary motor area, SMA) (Collomb-Clerc and Welter, 2015; Takakusaki, 2017), thus representing the cornerstone of MLR and key for sensorimotor integration (Mena-Segovia and Bolam, 2017). This role for the PNN in locomotor control has recently been supported by a study in five PD patients with GPi- and PPN-DBS that showed an increase in PPN neuronal activity during walking as compared to standing (Molina et al., 2020).

Within the basal ganglia, the striatum and its dopaminergic synapses are essential for motor learning and motor automaticity. Accordingly, the dopaminergic loss occurring in PD affects gait performances, especially when flexibility and adaptability in the gait pattern are required (Nutt et al., 1993, 2011b; Amboni et al., 2013; Fasano and Bloem, 2013; Santens, 2018).

The STN is ideally placed to regulate locomotion being directly connected with the SMA and projecting to the MLR structures (Nambu et al., 2002; Miocinovic et al., 2018). Accordingly, recent neurophysiological studies proved its role in locomotion control by assessing STN local field potentials (LFPs) in PD patients with advanced DBS devices (Rouse et al., 2011; Stanslaski et al., 2012). Time-frequency analysis of STN LFPs is altered in PD showing an *excessive* synchronization in the beta frequency band (13–35 Hz) and prolonged (>500 ms) beta-bursts (Oswal et al., 2013; Tinkhauser et al., 2017, 2018; Wang et al., 2018) in PD patients in meds-off (i.e., without medication) at rest. The neural activity of the STN during walking was mainly assessed as changes in beta synchronization as expressed by spectral power modulation. Fischer et al. (2018) showed a left-right alternating suppression of high-beta (20–30 Hz) spectral power in STN-LFPs of PD patients performing a visually guided stepping task while sitting and freely walking. Hell et al. (2018) reported suppression in high-beta power and bilateral oscillatory connectivity as well as a reduction in amplitude and duration of high-beta burst during gait as compared to rest. However, these findings are not consistent with the results of other studies that did not find STN beta suppression in freely moving PD patients (Quinn et al., 2015; Arnulfo et al., 2018). Quinn et al. (2015) reported similar STN beta power during lying, sitting, standing, and forward walking in 14 PD patients. We also found no difference in beta power during walking compared to sitting and standing in seven PD patients with STN-DBS (Arnulfo et al., 2018), but reported an interhemispheric decoupling (Arnulfo et al., 2018) and a frequency-shift of STN beta oscillations during gait (Canessa et al., 2020).

Less evidence is available for the GPi. Recent works suggested a role for this nucleus in locomotion inhibition (Aristieta et al., 2021). One study in patients with isolated dystonia (without gait abnormalities) and GPi-DBS studied LFPs during treadmill-gait. The authors showed a selective suppression of beta power during gait as compared to rest (Singh et al., 2011). In PD, instead, no changes of GPi beta power were found in five patients during walking as compared to standing (Molina et al., 2020), so that other frequency bands might be related to gait in GPi neurons.

The cortical contribution to gait control has also received great interest recently. Molecular imaging studies unveiled a diffuse cortical activation during gait (Jacobs and Horak, 2007; Yogev-Seligmann et al., 2008; Collomb-Clerc and Welter, 2015; Tard et al., 2015; Peterson and Horak, 2016). In particular, the primary motor cortex is relevant for gait adaptation that requires precise forelimb positioning to avoid obstacles or to change direction (Drew et al., 2002; Beloozerova et al., 2003; Dunin-Barkowski et al., 2006). The SMA is involved in balance control during locomotion and plays a role in the timing of the anticipatory postural adjustments (APA) during gait initiation (Richard et al., 2017). The posterior parietal cortex is necessary to plan and execute gait pattern adaptations by modifying the internal model of body representation during locomotion (McVea and Pearson, 2009). Reactive and predictive sensorimotor adjustments during gait are assumed to be ruled by internal models located in the cerebellum (Blakemore and Sirigu, 2003; Morton and Bastian, 2016), which is intimately connected with the temporoparietal cortex and the frontal cortices (Collomb-Clerc and Welter, 2015; Santens, 2018).

Studies on motor cortex activity during gait showed suppression of spectral power in alpha and beta frequency bands as well as changes in cortical connectivity during gait (Wang and Choi, 2020). In particular, alpha and beta band power suppression along with theta power increase in the sensorimotor cortex were documented in demanding walking tasks (e.g., obstacle avoidance) and likely reflect a greater cortical planning (Bulea et al., 2015; Nordin et al., 2019). In PD, one study showed increased interhemispheric synchronization across many frequency bands during walking as compared to healthy controls, thus suggesting a more prominent cortical involvement in locomotor control in PD patients (Miron-Shahar et al., 2019).

Finally, some studies have focused on specific gait alterations, such as freezing of gait (FOG), a sudden and transient disruption of the gait pattern (Nieuwboer and Giladi, 2013). Tard et al. (2015) performed a [18F]-fluorodeoxyglucose brain positron-emission tomography in PD patients showing FOG and documented a cortical hypometabolism as well as a dysregulation of the GPi, STN, and the MLR. At cortical level, one study showed an increase in theta power during FOG (Shine et al., 2014). Studies on STN LFPs showed instead higher beta frequency amplitude (Toledo et al., 2014; Hell et al., 2018) and an increase in alpha frequency entropy (Syrkin-Nikolau et al., 2017) in PD patients with FOG. Beta burst duration was found to be prolonged in PD patients with FOG (Anidi et al., 2018). However, being FOG an episodic phenomenon, it is crucial to assess electrophysiological alterations during actual freezing episodes. We recorded STN- and cortical LFPs in five PD patients with STN-DBS and FOG and found no difference in beta power, beta burst duration or interhemispheric STN coupling between effective walking and freezing episodes, but showed a low-frequency cortical-STN decoupling at the transition from normal walking into gait freezing, which resolved with the recovery of an effective gait pattern. Of note, these changes were found only on the side with less dopaminergic innervation, thus supporting a role for striatal dopamine in FOG (Pozzi et al., 2019).

Taken together these results suggest that altered neuronal oscillations in the supraspinal locomotor network are associated with the occurrence of gait disturbances in PD. Neural oscillations reflect fluctuations of local neuronal ensembles and their synchronization provide a mean for dynamic brain coordination (Buzsáki and Wang, 2012). Alterations in neuronal oscillation dynamics (i.e., timely synchronization and desynchronization) in the locomotor network may thus hamper locomotor control and result in gait impairments. This knowledge provides a rationale for treating gait disorders with neuromodulation tools, such as DBS, that allows retuning the activity of neural ensembles, even if distant from the implantation site, by means of modulation of neural networks dynamics.

## Clinical Assessment of Gait and Gait Disturbances in Parkinson’s Disease

The complex pathophysiology of gait disturbances in PD translates into great clinical variability that can vary from shuffle bradykinetic gait to dyskinetic pseudo-ataxic gait and include peculiar gait alterations, like FOG (Nieuwboer and Giladi, 2013) or reckless gait (Fasano and Bloem, 2013).

To treat gait disturbances in PD with DBS is important to recognize their specific clinical features (Giladi et al., 2002, 2013). To this end, a careful clinical history is essential (Fasano and Bloem, 2013; Nonnekes et al., 2019b), first to distinguish between *continuous* and *episodic* gait disturbances, as well as their relation with dopaminergic medications intake (Giladi et al., 2013). The use of instrumental aids (e.g., orthosis) or any other compensatory strategy should always be evaluated and can be particularly informative in patients with FOG (Fasano and Bloem, 2013). The risk of falls should also always be investigated and can easily be done by screening for a previous fall, which is a reliable predictor of new falls (Grimbergen et al., 2004). Further, the implanted nucleus, time to surgery and the active stimulation paradigm, as well as the permitted paradigms of stimulation by the implantable pulse generator (IPG), are essentials. Finally, in every patient with PD and STN-DBS with gait disturbances the lead location should critically be reviewed as even small misplacement might greatly impact the clinical outcome (Nickl et al., 2019).

The evaluation of gait cannot be separated from a complete neurological examination. It starts by assessing standing and postural abnormalities (broad base width, camptocormia, etc.) as well as the presence of dyskinesia or dystonia, which may differ in laying of standing position. Since DBS may also induce gait impairment, the clinical evaluation must be performed at least in stim-on and stim-off condition (i.e., with and without stimulation), although there is no consensus on the delay of the examination. Whenever possible, a prolonged suspension (up to 72 h) is recommended (Reich et al., 2016). Furthermore, we encourage to perform the clinical assessment in both meds-off and meds-on condition, especially in those PD patients still presenting motor fluctuations.

**FIGURE 1:**
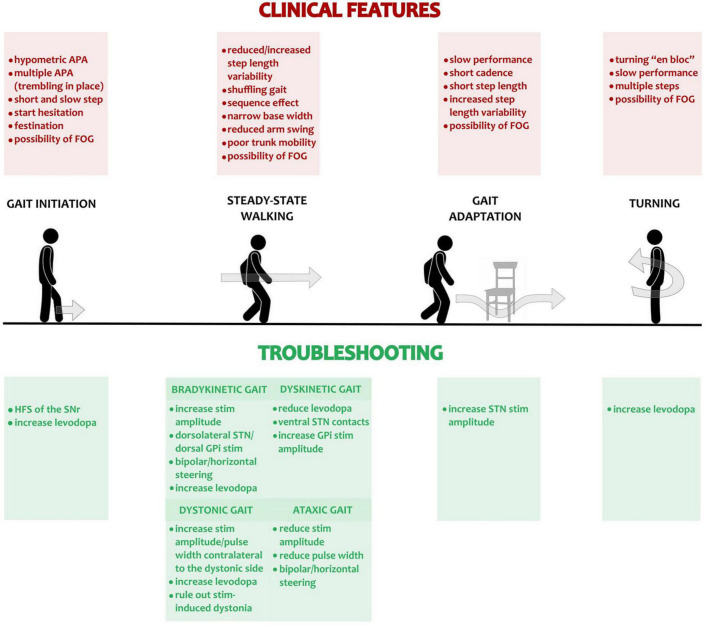
Gait disturbances in PD and possible troubleshooting with DBS. The main gait conditions (i.e., gait initiation, steady-state walking, gait adaptation and turning) are displayed. The top red panels list the most frequent pathological abnormalities occurring in PD patients in meds-off condition and assessed clinically or with a kinematic gait analysis. The green panels at the bottom list the adjustment in DBS programming and medications for troubleshooting the pathological changes of the different gait components. For steady-state, unperturbed linear walking, we addressed separately the possible adjustments in case of bradykinetic, dystonic, dyskinetic, or ataxic gait. APA, Anticipatory postural adjustments; FOG, freezing of gait; HSF, high frequency stimulation (i.e., >130 Hz); STN, subthalamic nucleus; GPi, Globus pallidus pars interna.

For clinical purposes, it may be useful to divide gait into four conditions: (1) gait initiation, (2) unperturbed steady-state walking, (3) turning, and (4) gait adaptation (Smulders et al., 2016; Figure 1). All these gait conditions can be described by specific biomechanical parameters (for review see Morris et al., 2001; Hof et al., 2005; Perry et al., 2010).

*Gait initiation* is the transition from quiet stance to steady-state walking. It is a highly challenging task for the balance control system and is of particular interest in the study of neural control of upright posture maintenance during whole-body movement (Delval et al., 2014). Gait initiation is characterized by APA, patterned muscular synergies (Farinelli et al., 2020) aiming to destabilize the antigravity postural set via misalignment between the center of pressure (CoP) and the center of mass (CoM) to generate a gravitational moment favoring CoM forward acceleration (Crenna and Frigo, 1991). The associated motor program seems to be centrally mediated (Palmisano et al., 2020b) with direct involvement of striatal dopamine (Petersen et al., 2012; Palmisano et al., 2020a). However, the contribution of the basal ganglia in gait initiation remains poorly known. This motor task also presents some methodological difficulties to be properly investigated in PD patients (Palmisano et al., 2020a,b). Subjects with PD usually have hypometric APA, with less weight shift than would be required to make an effective step (Burleigh-Jacobs et al., 1997; Jacobs et al., 2009). This translates clinically in a slower and shorter length of the first step as compared to healthy subjects (Rocchi et al., 2006; Jacobs et al., 2009), so that coordination of the movement pattern may not vary in PD (Rosin et al., 1997). The failure of APA is often associated with *start hesitation* (Giladi et al., 1992; Mancini et al., 2009), whereas multiple unsuccessful APA can occur with a subtype of FOG referred to as *trembling in place* (or *knee-trembling*) (Jacobs et al., 2009). Another pathological gait initiation pattern in PD, often associated with FOG, is *festination*, which is a rapid and progressive shortening of step length, accompanied by a compensatory increase in cadence (Nonnekes et al., 2019a).

*Unperturbed steady-state walking* refers to linear walking at preferred and constant speed on a flat surface. Even in the absence of biomechanical analysis, important spatiotemporal features of gait can be clinically evaluated, such as gait speed, cadence, steps variability, arm swing and limbs coordination. The step-length and -height for both feet separately can be also assessed. Unmedicated PD patients show bradykinetic gait with reduced step height and length (causing the typical *shuffling* gait), narrow base width, small step length variability with normal or increase cadence (Morris et al., 2001). PD patients can also show a progressive reduction of step length (i.e., sequence effect) as an expression of motor bradykinesia (Nutt et al., 2011a). The base width during walking is usually narrow in PD (Fasano and Bloem, 2013), while the step length variability may vary according to symptoms lateralization. This is usually more evident in the upper body, where bradykinesia is expressed by reduced arm swing and decreased range of motion of the trunk (Sterling et al., 2015). In case of great lateralization of the motor symptoms, a patient may present great stride-to-stride variability, an asymmetric reduction of arm swing, and poor range of motion of the trunk. Large gait variability is a dangerous alteration being associated with postural instability (Hausdorff et al., 1998; Hausdorff, 2005), which can lead to falls (Weiss et al., 2014). The presence of dyskinesia or dystonia might also alter the gait pattern with jerky movements of the limbs that may impair balance during walking. In this case, step length and gait velocity may be increased to reduce instability (Fasano and Bloem, 2013). The development of dyskinesia or dystonia during walking may be due to medication adjustments (e.g., levodopa-induced dyskinesia or meds-off dystonia) or be secondary to DBS itself (Krack et al., 1999; Baizabal-Carvallo and Jankovic, 2016).

*Turning* is one of the most frequent motor behaviors, taking place up to 100 times per hour (Mancini et al., 2016). Turing implies a modification of the gait pattern with asymmetrical steps and requires a dynamic adaptation of balance through coordinated movements of the trunk and limbs (Smulders et al., 2016). By asking the patient to turn it is possible to assess the mobility of the head, upper and lower part of the body, and the number of steps required. In PD, the physiological sequential movement of eyes-head-trunk-feet is lost in favor of an “en bloc” turning. This is characterized by a simultaneous onset of eyes, head, trunk, and leg movement (Crenna et al., 2007), which is slow and requires multiple steps (Smulders et al., 2016). Turning repetitively can elicit FOG (Reich et al., 2014), which appears most frequently at the end of a turn and affects the inner leg of the turn cycle (Spildooren et al., 2018).

*Gait adaptation* reflects the ability to modify the gait pattern and navigate the environment. While steady-state walking is a highly automatized process that requires minimal attention in healthy subjects (Patel et al., 2014), gait adaptation involves the activation of multiple brain areas (Hinton et al., 2019). Biomechanical studies have shown that PD patients are unstable and need more time to overcome an obstacle and hit it multiple times (Smulders et al., 2016). Gait adaptation can be particularly difficult in subjects with PD due to difficulties resisting external interference and task-switching (Amboni et al., 2013). This condition is known as “higher-level gait disorder” and presents typically with short cadence, short steps with marked step length variability, and FOG with poor response to walking aids (Nutt et al., 1993). Clinically, it may not be evident, but can be unmasked by obstacle crossing or dual-task walking. Patients should therefore be asked to walk through narrow passages (e.g., doors) or in a crowded space (Smulders et al., 2016; Pozzi et al., 2019). Another approach is to ask the patient to perform a cognitive task (e.g., backward counting) or a difficult motor task (e.g., carrying a tray) while walking. Under increased attentional demands, gait may become highly irregular or stop (i.e., “stops walking while talking” phenomenon) (Bloem et al., 2000; Hyndman and Ashburn, 2004). Alternatively, patients may neglect the onset of gait difficulties and focus on the cognitive task, thereby exhibiting reckless gait, a phenomenon more frequently observed in progressive supranuclear palsy (Ebersbach et al., 2013; Raccagni et al., 2019).

*Freezing of Gait* is an episodic and sudden interruption of the gait pattern with patients feeling the feet “glued to the ground” and the trunk is usually leant forward (Nieuwboer and Giladi, 2013). It occurs predominantly when the on-going locomotor pattern is interrupted (e.g., termination or initiation of gait), modulated (e.g., turning, obstacles navigation), or interfered (e.g., dual-task walking), particularly under time constraints (Bekkers et al., 2018). Focused attention and external stimuli (cues) may instead facilitate the overcoming of a FOG episode (Nieuwboer and Giladi, 2013).

With disease progression, the majority of PD patients develop FOG. A recent meta-analysis of 9,072 PD patients showed a weighted prevalence for FOG of 50.6% with a marked increase with years of disease (37.9% for ≤ 5 years vs. 64.6% for ≥ 9 years from diagnosis of PD) (Zhang et al., 2021). Patients predisposed to develop FOG usually show an altered locomotor pattern with increased step length variability and poor coordination (Nieuwboer and Giladi, 2013). FOG is associated with a high risk of falling and hospitalization (Bloem et al., 2004; Okuma et al., 2018). Falls likely occur because of weight-shifting impairments with inadequate scaling and timing of postural responses (Bekkers et al., 2018). As such, it represents a major determinant of poor quality of life in subjects with PD (Moore et al., 2007; Perez-Lloret et al., 2014). The pathophysiological mechanism leading to FOG abrupt onset remains largely unknown, but it likely involves transient derangements of the supraspinal locomotor network (Weiss et al., 2020).

FOG can be classified according to the medication state into meds-off FOG, meds-on FOG (i.e., induced by dopaminergic medication) and levodopa-resistant FOG, which persist after a supratherapeutic dose of levodopa (Nieuwboer and Giladi, 2013). Pseudo-on FOG is seen during seemingly optimal meds-on state, but which nevertheless improves with stronger dopaminergic stimulation (Espay et al., 2012).

**FIGURE 2:**
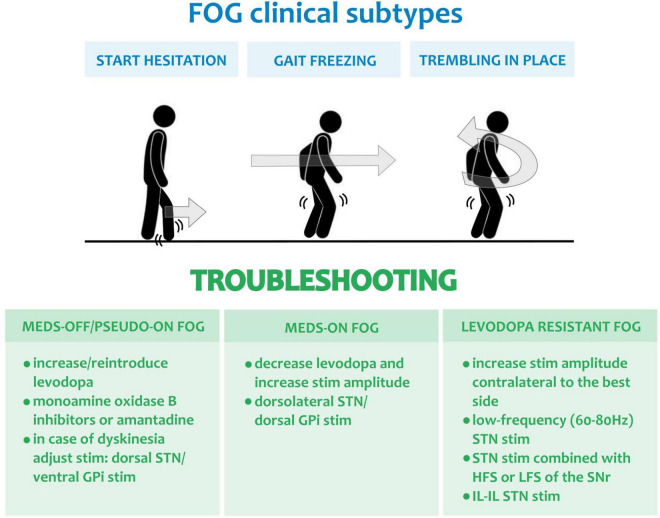
Freezing of gait and possible troubleshooting with DBS. The clinical subtypes of FOG are displayed in the top blue panel, namely: start hesitation, gait freezing and trembling in place. The green panels below report the stimulation and medication adjustments for troubleshooting the different forms of FOG and precisely: meds-off/pseudo-on FOG, meds-on FOG and L-Dopa resistant FOG. HSF, high frequency stimulation (i.e., >130 Hz); LSF, low frequency stimulation (i.e., <80 Hz); STN, subthalamic nucleus; GPi, Globus pallidus pars interna; SNr, Substatia Nigra pars reticulata; IL-IL, interleaved-interlinked (Karl et al., 2020).

FOG can be accompanied by additional though distinctive phenomena such as *start hesitation*, which is the inability in generating effective stepping at the beginning of walking, or *trembling in place*, which is shaking of the knees with the forefoot attached to the floor and the heel in the air (Nieuwboer and Giladi, 2013). Figure 2 shows the different clinical subtypes of FOG.

*Festination* represents a progressive increase in step cadence and gait speed with an excessive forward bending of the trunk that usually occurs during walking when approaching a destination. The pathophysiology of *gait festination* remains largely unclear, and it might be related to a defective production or processing of temporal cues at basal ganglia or cortical level (pre-SMA), respectively (Buhusi and Meck, 2005; Morris et al., 2008). Interestingly, a similar pathophysiological mechanism is shared by oral festination (Moreau et al., 2007; Ricciardi et al., 2016). A recent study advanced the hypothesis of a different subtype of festination in PD that derives from a postural abnormality (Nonnekes et al., 2019a). In this case, festination would emerge as a compensatory attempt to avoid falling due to the forward-leaning of the trunk and inappropriately small balance-correcting steps (Nonnekes et al., 2019a).

*Functional gait disorders* are characterized by symptoms not compatible with organically determined gait patterns and an inconsistent presentation with susceptibility to distraction (Baik and Lang, 2007; Araujo et al., 2019; Nonnekes et al., 2020). Functional movement disorders have been described also in subjects with PD following DBS (Breen et al., 2018; Maciel et al., 2021). A detailed discussion of functional movement disorders has been reported elsewhere (Edwards and Bhatia, 2012).

## Troubleshooting Gait Disturbances in Parkinson’s Disease Patients With Deep Brain Stimulation

In all PD patients with DBS that develop early gait disturbances reprogramming should be attempted as it can lead to marked clinical improvement. DBS reprogramming is a complex procedure that requires customization of stimulation delivery, based on the symptomatology, anatomy, pathophysiology, and pharmacological condition of each patient (Volkmann et al., 2006; Picillo et al., 2016; Hell et al., 2019; Koeglsperger et al., 2019). For these reasons, there is no fixed algorithm that could work for every patient. Still, some basic concepts may facilitate the reprogramming process that, for sake of clarity, can be broken down into (1) changes of the stimulation parameters (i.e., amplitude, frequency and pulse width), (2) changes of the stimulation location (e.g., by modifying the active contacts or steering the stimulation), (3) changes of the paradigm of stimulation (e.g., interleaving stimulation) (Dayal et al., 2017). Of note, the optimization of pharmacological therapy is also essential to achieve a lasting improvement. In this regard, we suggest performing a clinical evaluation in meds-on condition after reprogramming, which should be performed in meds-off condition whenever possible. We also encourage to wait up to 10 min to assess the efficacy of any stimulation change as the effects may not be instantaneous, especially if performed in meds-on. Finally, we strongly suggest including exercise and physical therapy in the treatment of PD patients with gait disturbances (Mak et al., 2017; Gilat et al., 2021).

### Gait Initiation Problems

Studies on the effects of DBS on gait initiation are few and with inconsistent results. Crenna et al. (2006) showed an improvement of both APA and the execution of the first step with unilateral and bilateral high frequency stimulation (HFS, i.e., > 130 Hz) of the STN, whereas Rocchi et al. (2012) reported an impairment of APA with bilateral STN- or GPi-DBS. The interesting observation that unilateral stimulation may improve bilateral symptoms led to the hypothesis of a “dominant” STN (Castrioto et al., 2011), which was documented in up to 50% of the patients with PD in one study (Rizzone et al., 2017).

STN-DBS and GPi-DBS did not improve compensatory stepping at gait initiation as compared to dopaminergic treatment (George et al., 2015). A selective improvement of gait disturbances at gait initiation was instead achieved with HFS of the SNr, which can be reached in some subjects with STN-DBS by selecting the most ventral contacts (Chastan et al., 2009; Scholten et al., 2017). This approach is still under investigation, but it might be used as a rescue strategy. Increasing dopaminergic medications can be also useful (Smulders et al., 2016) as levodopa showed to improve some APA (particularly the imbalance phase) and the stepping phases (Curtze et al., 2015; Palmisano et al., 2020a).

#### Troubleshooting

A summary is shown in Figure 1.

• Attempt HFS of the SNr (Figure 1; Chastan et al., 2009; Scholten et al., 2017).
• Adjust dopaminergic medications (e.g., increase levodopa) (Smulders et al., 2016; Figure 1).

### Unperturbed Steady-State Walking Problems

A bradykinetic gait may arise after DBS due to an excessive reduction of dopaminergic medications (Castrioto et al., 2014). The reduction of dopaminergic medication may also be responsible for the development of dystonic contraction during walking (Krack et al., 1999; Castrioto et al., 2013). On the other hand, the presence of levodopa-induced dyskinesia might alter profoundly the gait pattern with jerky movements of the limbs that impair balance and walking (Krack et al., 1999; Castrioto et al., 2013). This condition has recently been described as lower body dyskinesias, which can be due to the synergic effect of dopaminergic medications and STN-DBS (Cossu and Pau, 2017).

STN-DBS may also directly induce a bradykinetic worsening of gait through an inadvertent stimulation to the pallido-thalamic tract that runs in the zona incerta region located dorsally and medially to the STN dorsal Zona incerta (dZi) (Castrioto et al., 2013; Fleury et al., 2016). This side effect can also affect GPi-DBS for current spread in the *ansa lenticularis* (Castrioto et al., 2013; Baizabal-Carvallo and Jankovic, 2016). The inadvertent stimulation of pallidal projections to the PPN may be a cause lateralized bradykinetic gait too (Baizabal-Carvallo and Jankovic, 2016; Cossu and Pau, 2017). In rare cases, dystonic gait might be secondary to HFS within the STN or to an inadvertent chronic overstimulation with current spread to the corticospinal tract (Castrioto et al., 2013; Baizabal-Carvallo and Jankovic, 2016). More often, STN-DBS directly induces dyskinetic gait, which may develop with delay (up to several hours) after stimulation adjustments (Krack et al., 1999; Baizabal-Carvallo and Jankovic, 2016). Balance impairment can be instead induced by inadvertent stimulation of the red nucleus or cerebellar fibers (Felice et al., 1990). This side-effect is more commonly seen in patients with essential tremor (ET) and thalamic DBS (Reich et al., 2016) but can be present also in PD (Felice et al., 1990). Clinically, it manifests as an ataxic gait, with wide base width, high stride-to-stride and gait speed variability.

#### Troubleshooting

A summary of troubleshooting is shown in Figure 1.

• Bradykinetic gait

- Increase stimulation amplitude (Volkmann et al., 2006; Koeglsperger et al., 2019). In case of lateralized bradykinetic gait, the brain side contralateral to the worst hemibody should be addressed first (Figure 1).
- Try contacts at the dorsolateral margin of the STN (Figure 1; Herzog et al., 2004; Nickl et al., 2019). In case of GPi-DBS, a more dorsal stimulation is preferable (Figure 1; Bejjani et al., 1997; Rabin and Kumar, 2015; Baizabal-Carvallo and Jankovic, 2016; Au et al., 2020).
- In case of suspected inadvertent stimulation (e.g., dZi) use a bipolar configuration or the horizontal steering of the stimulation, if supported by segmented leads (Figure 1; Steigerwald et al., 2019). Still, an increase of the stimulation amplitude might be required to maintain sufficient control of motor fluctuations. In this case the use of an *anodic block* may be attempted (Figure 1; Valente et al., 2010).
- Adjust dopaminergic medications (e.g., increase levodopa; Figure 1) (Smulders et al., 2016).

• Dystonic gait

- Increase the stimulation amplitude or pulse-width contralateral to the dystonic side (Figure 1; Volkmann et al., 2006; Koeglsperger et al., 2019).
- Rule out the rare case of a stimulation-induced dystonia (Figure 1). Start with excluding pyramidal side-effects by reducing stimulation or performing a bipolar stimulation and beware that it may require a prolonged evaluation (up to few days). Eventually steer the stimulation outside the STN aiming to the dorsolateral border (Figure 1; Castrioto et al., 2013; Baizabal-Carvallo and Jankovic, 2016).
- Adjust dopaminergic medications (e.g., increase levodopa; Figure 1) (Smulders et al., 2016).

• Dyskinetic gait

- For STN-DBS, try dorsal contacts (Figure 1; Volkmann et al., 2006; Herzog et al., 2007; Aquino et al., 2019; Koeglsperger et al., 2019). In GPi-DBS an increase of the stimulation amplitude may suffice, otherwise test more ventral contacts (Figure 1; Bejjani et al., 1997; Krack et al., 1998; Rabin and Kumar, 2015; Baizabal-Carvallo and Jankovic, 2016; Au et al., 2020).
- Reduce dopaminergic medications (e.g., reduce levodopa) and eventually increase STN/GPi stimulation to preserve sufficient control of motor fluctuations (Figure 1).

• Ataxic gait

- Reduce the stimulation amplitude, but beware that this would come at the expense of the total electrical energy delivered (TEED) with likely worsening of motor fluctuations (Figure 1; Volkmann et al., 2006; Koeglsperger et al., 2019).
- Try short pulse width (Figure 1; Reich et al., 2015). This would allow for a more selective stimulation based on different neuronal chronaxies and increase the therapeutic window. An increase in stimulation amplitude of ∼0.5 mA/10 μs would be likely required.
- Use a bipolar configuration to limit the inadvertent current spread and TEED reduction (Figure 1). Still, an increase of the stimulation amplitude may be required to maintain sufficient control of motor fluctuations.
- With a segmented lead, the horizontal steering of the stimulation may allow an improvement of the symptomatology and can prevent inadvertent stimulation of nearby structures (Figure 1; Steigerwald et al., 2019). To this aim, the use of an *anodic block* may also be attempted (Valente et al., 2010).

### Turning Problems

STN-DBS has been reported to positively affect turning in PD by decreasing inter-segmental latencies (i.e., eye-head, eye-foot, and head-trunk) (Lohnes and Earhart, 2012). This benefit may be a specific effect of STN-DBS as dopaminergic medications improved turning during walking but not turning in place (Smulders et al., 2016). No data are available for GPi-DBS.

#### Troubleshooting

A summary is reported in Figure 1.

• No recommendation can be made due to the lack of evidence in the literature. We empirically suggest following the troubleshooting proposed for gait initiation, assessing the two hemibodies separately while the patient is asked to turn in place to the right and then to the left side (Figure 1).
• Adjust dopaminergic medications (increase levodopa to improve turning during walking; Figure 1) (Smulders et al., 2016).

### Gait Adaptation Problems

STN-DBS can improve dual-task gait with a selective effect on gait, but not on cognitive performances (Seri-Fainshtat et al., 2013; Chenji et al., 2017). Dopaminergic medications also improve the gait performances under attentional demands but also induce a less cautious behavior (Smulders et al., 2016; Raccagni et al., 2019). No data are available for GPi-DBS.

#### Troubleshooting

A summary is shown in Figure 1.

• No recommendation can be made due to the lack of evidence in the literature. We empirically suggest increasing the amplitude of STN stimulation to support gait in dual-tasking performances (Figure 1; Seri-Fainshtat et al., 2013; Chenji et al., 2017).

##### Freezing of Gait

The effect of DBS on FOG is debated. Some studies showed an improvement of FOG with HFS STN-DBS up to 4-year follow-up, especially for meds-off FOG (Pötter-Nerger and Volkmann, 2013; Vercruysse et al., 2014). A re-evaluation of the EARLYSTIM trial also showed that STN-DBS with best pharmacological treatment was superior to best pharmacological treatment alone in preventing FOG in PD patients at 3 years from surgery (Barbe et al., 2020). No benefit has instead been shown on meds-on FOG (Schlenstedt et al., 2017). Acute development of levodopa-resistant FOG after STN-DBS surgery has also been described and possibly related to the inadvertent stimulation of the pallidal projections to the PPN, which are located dorsally to the STN in the Forel field (Tommasi et al., 2007; Adams et al., 2011; Cossu and Pau, 2017).

The management of FOG with DBS has been assessed in a few studies with different approaches ranging from changes in stimulation parameters (Moreau et al., 2008; Fasano et al., 2011), location (Weiss et al., 2013), or paradigm (Karl et al., 2020).

Fasano et al. (2011) reported an improvement in FOG when reducing the STN-DBS amplitude of 50% for the best hemibody (i.e., contralateral to the leg with longer step length). This approach aims to restore gait coordination by reducing the step length variability, but it might not be applicable in all subjects as other parkinsonian symptoms might arise under reduced stimulation amplitude (Meoni et al., 2019).

Moreau et al. (2008) achieved a remarkable improvement of FOG by reducing the frequency of stimulation to 80 Hz (low-frequency stimulation, LFS). The effect of LFS on human locomotion is not entirely clear, but it may be related to the modulation of STN fibers projecting to the PPN (Xie et al., 2017). LFS seems especially effective in PD patients who develop FOG with HFS, regardless of medication condition (Xie et al., 2017). A long-lasting positive effect of LFS can be expected in PD patients with more anterior stimulation of the STN (Zibetti et al., 2016). This can be achieved with horizontal current steering in subjects implanted with segmented leads (Steigerwald et al., 2019). However, in many cases, the benefit is only temporary and parallels a worsening of akinetic-rigid signs (Ricchi et al., 2012). Of note, unlike amplitude and frequency changes, increasing pulse-width is usually not associated with FOG improvement and might induce gait deterioration by increasing the current spread (Hui et al., 2020). Short pulse-width also showed no significant changes on FOG, while it was associated with an improvement of speech (Seger et al., 2021).

When changes in stimulation parameters are ineffective, a different stimulation location can be tested. Weiss et al. (2013) first reported a long-term improvement in FOG by combining STN- and SNr-HFS. Subsequently, FOG improvement was reported also for STN-HFS and SNr-LFS (Valldeoriola et al., 2019). Technological advances (e.g., vertically current steering and multiple independent current control) now more easily allow for such configurations (Andreasi et al., 2020), the efficacy of which is yet to be confirmed with large studies.

Finally, an improvement of FOG can be achieved by changing the stimulation paradigm. In particular, Karl et al. (2020) showed in a preliminary report in 25 PD patients a substantial benefit of interleaved-interlinked (IL-IL) STN-DBS on gait and FOG. This stimulation is a monopolar interleaved, overlapping, LFS of the STN generating a large stimulation field with peripherical LFS and central HFS (overlapping area).

Limited evidence is available for GPi-DBS in the management of FOG. GPi-DBS can improve FOG in meds-off condition with a sustained effect up to 4 years (Rodriguez-Oroz et al., 2005; Pötter-Nerger and Volkmann, 2013). However, in the meds-on condition, the improvement of GPi-DBS on FOG was limited to 1-year (Volkmann et al., 2004). An observational study specifically evaluating the effect of GPi-DBS on FOG in patients with PD is ongoing (NCT03227250) and more reports on this topic are encouraged.

#### Troubleshooting

A summary of troubleshooting is shown in Figure 2.

• Meds-off FOG and pseudo-on FOG:

- Increase dopaminergic medications and consider prescribing monoamine oxidase B inhibitors or amantadine (Figure 2; Fasano and Lang, 2015; Nonnekes et al., 2015). In case of monotherapy with dopamine agonists, consider reintroducing levodopa. In the event of troublesome dyskinesia, an adjustment of stimulation might be needed: for STN-DBS more dorsal contacts should be tried (Volkmann et al., 2006; Herzog et al., 2007; Aquino et al., 2019; Koeglsperger et al., 2019), while for GPi-DBS an increase of the stimulation amplitude or more ventral contacts should be tested (Figure 2; Bejjani et al., 1997; Krack et al., 1998; Rabin and Kumar, 2015; Baizabal-Carvallo and Jankovic, 2016; Au et al., 2020).

• Meds-on FOG

- Reduce dopaminergic medications (Fasano and Lang, 2015; Nonnekes et al., 2015) and consider increasing the amplitude of stimulation (Figure 2). For STN-DBS dorsolateral contacts should be selected (Volkmann et al., 2006; Herzog et al., 2007; Aquino et al., 2019; Koeglsperger et al., 2019), while dorsal contacts are preferrable for GPi-DBS (Figure 2; Bejjani et al., 1997; Krack et al., 1998; Rabin and Kumar, 2015; Baizabal-Carvallo and Jankovic, 2016; Au et al., 2020).

• Levodopa-resistant FOG:

- Reduce the STN-DBS amplitude contralateral to the best hemibody (Fasano et al., 2011) or bilaterally in case of stimulation-related FOG in patients with GPi-DBS (Figure 2; Sketchler and Shahed, 2019).
- Try STN-DBS with low frequency (60–80 Hz; Figure 2) (Moreau et al., 2008). An increase in stimulation amplitude may be needed to maintain a comparable TEED (Hui et al., 2020).
- Combine STN with SNr-HFS (Weiss et al., 2013) or -LFS (Figure 2; Valldeoriola et al., 2019).
- Test IL-IL STN-DBS (Figure 2; Karl et al., 2020).

### Functional Gait Disorders

The occurrence of functional movement disorders after DBS is not common, but it has been reported (Breen et al., 2018; Maciel et al., 2021).

#### Troubleshooting

• Functional gait disorders are not organically determined, therefore should be treated with diagnostic counseling. Changes in DBS parameters are not suggested, but a careful reevaluation of the stimulation should be performed anyhow as suboptimal programming (e.g., inadvertent stimulation of the anterior part of the STN in STN-DBS) may worsen non-motor symptoms (Castrioto et al., 2014; Petry-Schmelzer et al., 2019).

## Lead Revision and Alternative Deep Brain Stimulation Target for Gait Disturbances: When Reprogramming Is Not Enough

Despite optimized pharmacological and stimulation treatments gait disturbances might still determine a significant burden for some PD patients. When all reprogramming options have been exploited, a surgical revision of the leads can be considered. Beside suboptimal lead placement, a supportive criterion to lead revision is the presence of an optimal levodopa response. In these patients, we reported a marked motor improvement after lead repositioning, even years after DBS surgery. However, the main improvement was achieved on rigidity or tremor (Nickl et al., 2019). As such, the repositioning of the lead in case of gait disturbances must be critically and individually discussed.

Treatment-resistant gait disturbances also promoted the investigation of alternative targets for DBS, such as the MLR or the field of Forel.

Stefani et al. (2007) first reported a remarkable improvement of gait and axial symptoms in six PD patients with combined STN-HFS and PPN-LFS (i.e., 25 Hz) at 6-month follow-up. This finding was initially confirmed (Moro et al., 2010; Peppe et al., 2010; Welter et al., 2015), but more recent studies have reported only a marginal benefit for PPN-DBS, limited to FOG (Ferraye et al., 2010), and only up to 3 months post-intervention (Wang et al., 2017; Yu et al., 2020). Caution is needed when interpreting these results due to the small and heterogeneous sample size as well as to the variability in the surgical placement of the leads. Some controversy remains also on unilateral vs. bilateral PPN-DBS as randomized, double-blinded studies showed FOG improvement with unilateral stimulation only (Rahimpour et al., 2020). It is still unclear which stimulation frequency should be preferred as benefits were reported for a wide range spanning from 15 to 130 Hz (Rahimpour et al., 2020). Furthermore, less is known on the effect of PPN-DBS on non-motor, which might be relevant as PPN stimulation can impact alertness and sleep (Sharma et al., 2018).

Encouraging results on FOG in PD have also been reported for stimulation of another MLR structure, namely the CN (Goetz et al., 2019). A prospective pilot trial of directional CN-DBS is ongoing (NCT04218526). An alternative promising target has recently been proposed by Rocha et al. (2021), who reported a marked and lasting motor improvement with amelioration of PIGD in 13 PD patients with Filed of Forel DBS.

Finally, a combined GPi- and PPN-DBS was investigated in a recent study that showed an improvement of FOG in three out of five PD patients at 6-month but not 1-year follow-up (Molina et al., 2021). The subsequent attempt in the same patients of an adaptive DBS with the stimulation triggered by an increase in power of the 1–8 Hz band from the PPN region was not successful (Molina et al., 2021). Despite the negative result, this study is of great value as it highlights the relevance that an accurate and stable neurophysiological biomarker of gait may have in advancing the treatments of gait impairment in PD. To this end, the management of non-neuronal artifacts will be also essential (Neumann et al., 2021; Thenaisie et al., 2021).

## Concluding Remarks and Future Perspective

Gait disturbances are among the most relevant determinates of poor quality of life in PD and remain a therapeutic challenge, representing a cause of dissatisfaction after DBS surgery. While chronic gait impairments may be related to disease progression and require a combined and multidisciplinary therapy, early gait disturbances arising in the first 3 years after surgery may be secondary to treatable causes. In all these patients reprogramming of DBS should be attempted as it can lead to marked clinical improvement.

Advances in the neurophysiological understanding of gait control will soon lead to the development of novel DBS devices that can monitor the neuronal correlates of gait or its alterations and possibly adapt the stimulation delivery accordingly (Little and Brown, 2020; Gilron et al., 2021). Meanwhile, the optimization of DBS parameters needs to be performed clinically and it is based on proper classification of the gait disturbances. The clinical characterization of gait disturbances gives insight into their pathophysiological mechanism and can guide reprogramming, which has led to a marked improvement of the clinical outcome in a considerable number of PD patients. Alternative brain targets for DBS remain investigational but might be used as rescue therapy in selected cases.

## Author Contributions

NP and IUI contributed to study design and planning. NP, CHP, PC, and MR contributed to literature analysis and search. CLP, JV, and IUI contributed to data interpretation and collection of funds. NP drafted the manuscript. NP and CHP prepared the figure. PC, MR, CLP, JV, and IUI critically reviewed and contributed to preparation of the manuscript. All authors contributed to the article and approved the submitted version.

## Conflict of Interest

The authors declare that the research was conducted in the absence of any commercial or financial relationships that could be construed as a potential conflict of interest.

## Publisher’s Note

All claims expressed in this article are solely those of the authors and do not necessarily represent those of their affiliated organizations, or those of the publisher, the editors and the reviewers. Any product that may be evaluated in this article, or claim that may be made by its manufacturer, is not guaranteed or endorsed by the publisher.
